# Lactation and resource limitation affect stress responses, thyroid hormones, immune function, and antioxidant capacity of sea otters (*Enhydra lutris*)

**DOI:** 10.1002/ece3.4280

**Published:** 2018-07-25

**Authors:** Sarah M. Chinn, Daniel H. Monson, M. Tim Tinker, Michelle M. Staedler, Daniel E. Crocker

**Affiliations:** ^1^ Department of Biology Sonoma State University Rohnert Park California; ^2^ U.S. Geological Survey Alaska Science Center Anchorage Alaska; ^3^ U.S. Geological Survey Western Ecological Research Center Long Marine Laboratory Santa Cruz California; ^4^ Monterey Bay Aquarium Monterey California

**Keywords:** lactation, resource limitation, sea otter, trade‐offs

## Abstract

Lactation is the most energetically demanding stage of reproduction in female mammals. Increased energetic allocation toward current reproduction may result in fitness costs, although the mechanisms underlying these trade‐offs are not well understood. Trade‐offs during lactation may include reduced energetic allocation to cellular maintenance, immune response, and survival and may be influenced by resource limitation. As the smallest marine mammal, sea otters (*Enhydra lutris*) have the highest mass‐specific metabolic rate necessitating substantial energetic requirements for survival. To provide the increased energy needed for lactation, female sea otters significantly increase foraging effort, especially during late‐lactation. Caloric insufficiency during lactation is reflected in the high numbers of maternal deaths due to End‐Lactation Syndrome in the California subpopulation. We investigated the effects of lactation and resource limitation on maternal stress responses, metabolic regulation, immune function, and antioxidant capacity in two subspecies of wild sea otters (northern: *E. l. nereis* and southern: *E. l. kenyoni*) within the California, Washington, and Alaska subpopulations. Lactation and resource limitation were associated with reduced glucocorticoid responses to acute capture stress. Corticosterone release was lower in lactating otters. Cortisol release was lower under resource limitation and suppression during lactation was only evident under resource limitation. Lactation and resource limitation were associated with alterations in thyroid hormones. Immune responses and total antioxidant capacity were not reduced by lactation or resource limitation. Southern sea otters exhibited higher concentrations of antioxidants, immunoglobulins, and thyroid hormones than northern sea otters. These data provide evidence for allocation trade‐offs during reproduction and in response to nutrient limitation but suggest self‐maintenance of immune function and antioxidant defenses despite energetic constraints. Income‐breeding strategists may be especially vulnerable to the consequences of stress and modulation of thyroid function when food resources are insufficient to support successful reproduction and may come at a cost to survival, and thereby influence population trends.

## 1 INTRODUCTION

Lactation is the most energetically expensive portion of the mammalian reproductive cycle (Hanwell & Peaker, 1977). Mean daily caloric intake can be 66–200% greater, and peak energy expenditure may be 2.5–5 times higher in lactating females compared with nonlactating conspecifics (Gittleman & Thompson, 1988). During lactation, behavioral changes often coincide with increased energetic demands. For example, females may significantly increase duration and frequency of foraging events in order to balance increased energetic costs (Clutton‐Brock, Iason, Albon, & Guinness, 1982; Stebbins, 1977). In addition to increasing energy intake, reproducing females are able to catabolize body tissue as a nutrient source (Millar, 1977; Randolph, Randolph, Mattingly, & Foster, 1977).

Species are frequently classified along a continuum of reproductive strategies ranging from capital (rely mostly on stored energy during reproduction) to income breeders (rely mostly on acquired energy during reproduction), depending on the importance of stored body reserves in relation to reproductive effort (Jönsson, 1997; Stearns, 1992). Yet even for income breeders, for which body reserves provide only a small proportion of reproductive expenditure, reserves may still be important to supplement physiological limits at the periods of peak energy investment (Tyler, 1987). Foraging success in relation to reproduction may be a key determinant of the magnitude of reproductive effort (Clutton‐Brock et al., 1982; Crocker, Williams, Costa, & Le Boeuf, 2001).

For many species, the high energetic demands required for reproduction necessitate various life‐history trade‐offs. For example, increased energetic allocation toward a current reproductive event may come at a cost to future survival or reproduction (Stearns, 1992; Williams, 1966). These trade‐offs may involve ecological (e.g., increased risk of predation due to more time spent foraging) and physiological processes (e.g., competing cellular functions) (Speakman, 2008; Zera & Harshman, 2001). Trade‐offs during lactation may drive reductions in energy allocation to cellular maintenance and somatic function, and thereby reduce future reproduction and survival (Zera & Harshman, 2001). Direct costs arise from the energetic demands of the reproductive event, such as dramatic increases in nutrients for milk production (Hanwell & Peaker, 1977; Speakman, 2008). Indirect costs can be compensatory, where the female makes optional allocation trade‐offs in other components of its physiology such as altering thermoregulation (Trayhurn, 1989) and immunosuppression (Peck, Costa, Crocker, & Goldbogen, 2015) to increase the energy available for parental investment. Indirect costs can also be obligatory; for example, reproductive events may decrease maternal bone mass (Miller & Bowman, 2004) and increase oxidative stress (Sharick, Vazquez‐Medina, Ortiz, & Crocker, 2015; Wiersma, Selman, Speakman, & Verhulst, 2004). In all of the above examples, reductions in the environmental availability of food resources (henceforth, resource limitation) will tend to exacerbate trade‐offs between competing life‐history processes (Boggs & Ross, 1993; Cotter, Simpson, Raubenheimer, & Wilson, 2011; Martin, 1987).

Fluctuations in environmental and state conditions have the potential to affect homeostasis of wild animals. Energy demands of reproduction may illicit a stress response by activating the hypothalamic–pituitary–adrenal (HPA) axis to release glucocorticoid (GC) hormones (e.g., corticosterone and cortisol). Upon activation of the HPA axis, most species release predominant quantities of a single glucocorticoid—either cortisol or corticosterone, and smaller quantities of the other (Palme, Rettenbacher, Touma, El‐Bahr, & Möstl, 2005). Within mammals, cortisol is the predominant GC in humans, ruminants, and carnivores (Palme et al., 2005), but many mammals also release biologically significant amounts of corticosterone (Koren et al., 2012). GCs have expansive physiological consequences on metabolism, reproduction, and immune function (Ensminger, Somo, Houser, & Crocker, 2014; Maule, Schreck, & Kaattari, 1987; Tilbrook, Turner, & Clarke, 2000). GCs are known to be immunosuppressive (Råberg, Grahn, Hasselquist, & Svensson, 1998; Sheldon & Verhulst, 1996) and have been hypothesized as a proximate mediator of allocation trade‐offs between reproduction and immune function. During periods when GC levels are increased to support energy mobilization for reproduction, there may be a reduced ability to respond to additional stressors. Under conditions of chronic stress, an additional acute stressor can elicit the HPA axis to respond in either of two ways: facilitation or acclimation (Dickens & Romero, 2013; Figueiredo, Bodie, Tauchi, Dolgas, & Herman, 2003; Pitman, Ottenweller, & Natelson, 1988; Romero, 2004). The literature describing downregulation of GC activity (i.e., gene regulation, receptor availability and resistance, hypocortisolism) in response to an acute stressor under conditions of chronic stress are robust in the biomedical field (Heim, Ehlert, & Hellhammer, 2000; McEwen, 2000; Miller, Chen, & Zhou, 2007; Juster , McEwen, & Lupien, 2010). Fewer studies have assessed the effects of chronic stress on HPA sensitivity in wild animals, especially mammals, some of which show that under chronic stress (e.g., population decline, density, and predation pressure) some species respond to acute stressors by facilitation (Boonstra, Hik, Singleton, & Tinnikov, 1998; Clinchy, Zanette, Boonstra, Wingfield, & Smith, 2004; Boonstra, 2013; Dantzer et al., 2013). Whereas others exhibit decreased basal GC levels, and/or less GC is released in response to an acute stressor (Harlow, Lindzey, Sickle, & Gern, 1992; Krause, Dorsa, & Wingfield, 2014; Rich & Romero, 2005; Wingfield & Mukai, 2009). Therefore, chronic stress may not always be identifiable by upregulated GC concentrations. The intricacies of the stress response in wildlife do not allow for consistent predications of response outcomes, rather a best approach to identifying chronic stress is to measure multiple features of GC regulation and changes in those responses (Dickens & Romero, 2013).

Glucocorticoids interact with thyroid hormones (thyroxine, T_4_; and triiodothyronine, T_3_), which are regulators of metabolic pathways. In response to a stressor, such as nutrient limitation, GCs tend to increase to mobilize energy stores (McMahon, Gerich, & Rizza, 1988; Sapolsky, Romero, & Munck, 2000) and thyroid hormones tend to decrease to reduce energy expenditure (Oppenheimer et al., 1987). GCs reduce levels of circulating thyroid hormone by suppressing release of thyroid‐stimulating hormone and inhibiting enzymatic deiodination of T_4_ into T_3_, the more physiologically active form (Oppenheimer et al., 1987). Additionally, upregulated GCs increase concentrations of reverse T_3_ (rT_3_), an inactive form of the hormone which functions as a thyroid receptor blocker (Charmandari, Tsigos, & Chrousos, 2005). Reductions in thyroid hormones may directly decrease maternal energy expenditure but at the expense of thermoregulation and activity.

Oxidative stress may be an obligatory cost of reproduction, and when present, restricts energy allocation to other important life stages (Costantini, 2008; Dowling & Simmons, 2009). Although oxidative stress may regulate life‐history trade‐offs, its effects on different tissues during reproduction are still poorly understood (Speakman et al., 2015). Oxidative stress occurs when reactive oxygen species (ROS) production overwhelms the body's antioxidant defense systems (Monaghan, Metcalfe, & Torres, 2009). Life stages with increased energy investment associated with reproduction, such as lactation, may lead to overproduction of ROS (Alonso‐Alvarez et al., 2004; Balaban, Nemoto, & Finkel, 2005), which may result in the depletion of antioxidant defenses and progress to oxidative damage and inflammation, especially under conditions of nutrient limitation (Mårtensson, 1986; Sharick et al., 2015). Conversely, increased foraging during lactation may enhance availability of exogenous antioxidants, specifically if the food sources contain high levels of antioxidants that can act efficiently in vivo, helping mitigate the enhanced oxidative stress associated with high rates of energy expenditure during lactation (Beaulieu & Schaefer, 2013; Schinella, Marín, de Alaniz, de Buschiazzo, & Tournier, 1999).

**Figure 1:**
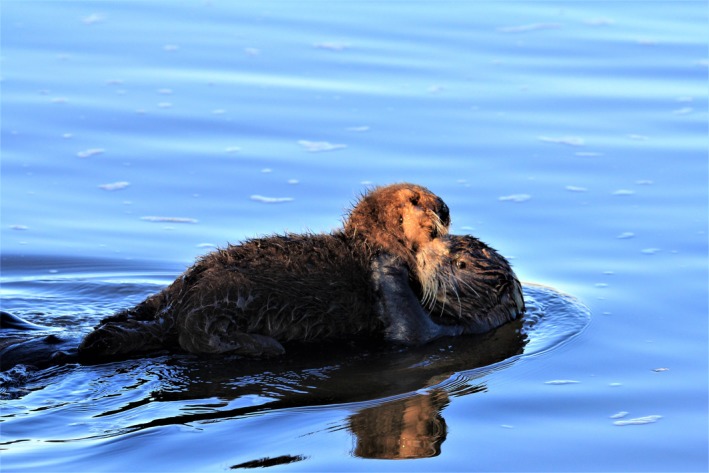
Sea otter (*Enhydra lutris*) female with small pup. Sea otters are the smallest marine mammal spanning the nearshore intertidal habitats of the North Pacific

Sea otters (*Enhydra lutris*) (Figure 1) provide an excellent study system for examining life‐history trade‐offs and physiological responses to the energetic demands of lactation, particularly in the context of resource limitation. Female sea otters give birth to a single pup at approximately 1‐year intervals—annual birth rates vary from 0.84 to 1.07 and appear to be relatively invariant across populations (Bodkin, Mulcahy, & Lensink, 1993; Monson, Estes, Bodkin, & Siniff, 2000; Riedman, Estes, Staedler, Giles, & Carlson, 1994; Tinker et al., 2017). The consistency of birth rates, even during periods of reduced food abundance, is thought to represent a form of bet‐hedging that is adaptive given temporally unpredictable prey resources (Monson, Estes, et al., 2000): females initiate pregnancy irrespective of their current condition in order to capitalize on possible future increases in prey abundance occurring during the ~6 month gestation period and before parturition. Only females provide pup care, and the average pup dependency period is 6 months (Monson, Estes, et al., 2000; Riedman et al., 1994). The elevated energetic costs associated with lactation necessitate significant increases in maternal foraging activity, especially during the latter half of the lactation period (Esslinger, Bodkin, Breton, Burns, & Monson, 2014; Staedler, 2011; Thometz, Staedler, et al., 2016). Necropsies of pregnant females found that they appear to maximize fat deposition during gestation (Chinn et al., 2016), suggesting that they may accrue some reserves prior to parturition. However, once sexually mature, female sea otters are perpetually alternating between pregnancy and lactation, with little allowance for energetic recovery, and therefore provide an excellent example of an “income” strategist.

Sea otter pups suckle throughout the dependency period, but also begin to receive solid prey after 4 weeks, and by the end of lactation may consume up to a third of the prey captured by their mothers (Riedman et al., 1994). Respirometry studies indicate that a mother's daily energy consumption increases 85–110% over nonlactating periods (Thometz, Kendall, Richter, & Williams, 2016; Thometz, Tinker, Staedler, Mayer, & Williams, 2014). Despite increasing their foraging effort to as much as 60% of each 24‐h period (Esslinger et al., 2014; Thometz, Staedler, et al., 2016), females in resource‐limited environments are often emaciated by the end of lactation. The extreme energetic demands elicited by lactation and pup‐rearing, in addition to exogenous stressors such as resource limitation (Tinker, Doak, & Estes, 2008; Tinker et al., 2006), can increase risk of metabolic collapse and mortality, a phenomenon known as “End‐Lactation Syndrome” (ELS) (Chinn et al., 2016).

Our objective was to investigate the physiological dimensions of the life‐history trade‐offs made by sea otters, in order to understand how an extremely energy‐limited mammal is able to adjust to the increased energetic demands of lactation. Sea otters are well suited for this analysis because their history of overexploitation and subsequent population recovery has created a natural experiment where populations exist with widely varying levels of resource availability. Sea otters were hunted nearly to extinction during the maritime fur trade in the 18th and 19th centuries, which left a fraction of the original population scattered throughout their range in 12 remnant populations (Kenyon, 1969). Following protection in 1911 and with the assistance of translocations into areas of SE Alaska, British Columbia, and Washington in the 1970's (Jameson, Kenyon, Johnson, & Wight, 1982), these remnant populations began to grow and reoccupy habitats that had not had otters in some cases for over 100 years. Sea otters are a keystone species having strong direct and indirect impacts on the nearshore food webs of which they are a part (Breen, Carson, Foster, & Stewart, 1982; Duggins, Simenstad, & Estes, 1989; Estes & Duggins, 1995; Estes & Palmisano, 1974). As a result, the invertebrate prey populations in unoccupied habitats increased dramatically with standing stocks often orders of magnitude greater than in areas with long‐established populations (Estes & Duggins, 1995; Estes & Palmisano, 1974). Currently, areas of unoccupied habitat still exist with adjacent sea otter populations living with abundant prey as they expand their range (Bodkin & Udevitz, 1999; Estes, 1990). In contrast, other populations expanded and recovered to pre‐exploitation (i.e., equilibrium) densities as early as the 1950s where food became an important limiting resource (Coletti, Bodkin, Monson, Ballachey, & Dean, 2016; Estes, 1990; Kenyon, 1969; Monson, Estes, et al., 2000). Most recently, a top‐down‐driven decline in the western Aleutians sea otter population caused by killer whale predation has again created low‐density sea otter populations existing with nonlimiting prey resources (Estes, Tinker, Williams, & Doak, 1998; Williams, Estes, Doak, & Springer, 2004). This spatio/temporal pattern of populations existing with various levels of food resources has been used to understand demographic factors controlling sea otter population dynamics in the past (Monson, Estes, et al., 2000). In addition, it has allowed researchers the ability to develop tools to assess population status relative to food resources including assessments of sea otter body size and condition, direct measures of energy recovery rates and time‐activity budgets, and dietary diversity (Bodkin, Monson, & Esslinger, 2007; Monson & Bowen, 2015; Newsome et al., 2015; Tinker, Bentall, & Estes, 2008).

Here, we explore how lactation affects maternal immune function, energy regulation, and antioxidant capacity across several subpopulations of wild sea otters existing at varying levels of resource availability. We compared a suite of blood analytes between lactating and nonlactating sea otters and subspecies, and compared populations for evidence of effects of resource limitation. We predicted changes in GC response to the acute stress of capture in females that were already experiencing chronic stress due to lactation. We predicted allocation trade‐offs due to energetic demands of lactation to suppress immune function and metabolism, and decreased antioxidant availability to combat oxidative damage.

## 2 MATERIALS AND METHODS

### 2.1 Sample collection

**Figure 2:**
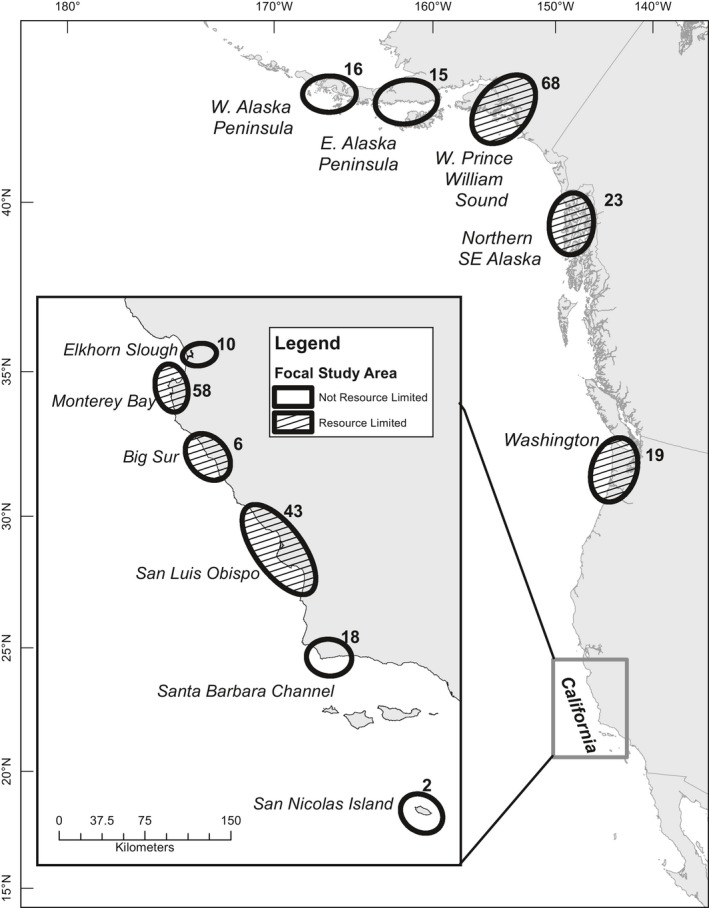
All subpopulations of sea otters sampled from California, Washington, and Alaska, USA. Circles with hatched fill were considered resource limited at the time of sampling. Number of otters sampled in each are listed above/to the right of each circle. Resource limitation status was assessed based on population density, recent trends, diet diversity, and biomass intake rates while foraging: for details, refer to Newsome et al. (2015)

Wild, reproductively active adult female sea otters were captured along the California, USA, coastline from 1999 to 2016, Alaska, USA, from 2006 to 2011 and Washington, USA, in 2011 with floating tangle nets, dip nets or by rebreather divers using modified Wilson traps (Ames, Hardy, & Wendell, 1986). The sample (*n* = 278) included 137 individuals from southern subspecies in California (*E. l. nereis*, subsequently referred to as SSO) and 141 from the northern subspecies in Alaska and Washington (*E. l. kenyoni*, subsequently referred to as NSO) (Figure 2). Of these, 112 females were captured with a dependent pup (lactating) and 166 were reproductively mature, nonlactating females. Lactating females were kept with their pups until anesthetized, and pups were returned to their mother prior to the female coming out of anesthesia and subsequently released together to avoid separation. Captured sea otters were held for variable times in wooden crates before anesthetization and sample collection. Otters were anesthetized via an intramuscular injection using a combination of 0.22–0.33 mg/kg fentanyl citrate and 0.07–0.11 mg/kg midazolam hydrochloride (Monson, McCormick, & Ballachey, 2001; Tinker et al., 2006). Morphometrics, nutritional state, and reproductive status were recorded, and blood was collected at a single event. A premolar tooth was collected for aging via analysis of cementum annuli (Bodkin et al., 1993; Matson's Lab, Milltown, MT, USA). When a cementum age was not available, age was determined by all physical characteristics including condition of teeth and coloration of pelage. Precise capture times were recorded for sea otters caught with Wilson traps and dip nets, and preanesthesia hold times were calculated. Precise capture times with tangle nets were not known but hold times for these sea otters were calculated from time of removal from net (which also represents a stressful event). Hold times were only recorded and available for a subset of the captures (*n* = 186).

Following sedation, whole blood was obtained from the jugular vein with 21 or 19 g butterfly winged blood collection sets into vacutainers and centrifuged (5–15 min at 2500–3700 RPM) to separate serum or plasma, then frozen at −80°C until analysis.

Subpopulations sampled were classified as resource‐limited or resource‐abundant, based on patterns of covariation in key metrics known to be responsive to resource abundance: population density and trends; diet diversity and prey recovery rates from foraging observations; relative body condition; and time‐activity budgets (Monson & Bowen, 2015; Newsome et al., 2015; Tinker, Doak, et al., 2008, 2017) (Figure 2; for additional details see Appendix Table S1).

### 2.2 Sample analysis

All analytes were measured in duplicate using commercially available assay platforms according to manufacturer's specifications. Cortisol and thyroid (total T_3_, total T_4_, and rT_3_) concentrations were measured using hormone specific I^125^ radioimmunoassays (MP Biological, Santa Ana, CA, USA, and ALPCO, Salem, NH, USA, respectively). Total antioxidant capacity (TAC) and corticosterone concentrations were measured using fluorometric and colorimetric enzyme‐immunoassays, respectively (Cayman Chemicals, Ann Arbor, MI, USA). Adaptive immune function was measured in two classes of total immunoglobulins, IgG, and IgM, using antibody‐sensitized microsphere microagglutination assays (Thermo Fisher Scientific, Waltham, MA, USA).

Samples were run “neat” (undiluted) except for corticosterone (diluted 1:15, with EIA buffer), IgM and IgG (diluted 1:100 000 or 1:2400, with dilution buffer) and TAC (diluted 1:10, with EIA buffer) because sample values were off the standard curve. All assay platforms were validated for use in sea otters. Serially diluted samples yielded curves that were parallel to the standard curves and between 89% and 106% of added standards were recovered from samples. The mean coefficient of variation was ≤5% for all analytes.

### 2.3 Statistical analysis

Data analyses were performed with JMP 13 Pro (SAS Institute, Cary, NC, USA). General linear models (GLM) were used to assess the effects of subspecies, maternal age, lactation status, resource limitation, and the interaction of lactation status with resource limitation on analyte concentrations. To examine associations between the markers, we used a GLM ANCOVA with an interaction term for lactation status and the independent variable. To address the ability to mount an acute stress response, sea otters with known time prior to anesthesia (duration between capture time or first observation in tangle net, until anesthesia) were plotted against GC values. If hold times exceeded 60 min, those individuals were excluded from analysis to account for clearance of GC from the initial stress response. While the half‐life of endogenous GCs in sea otters is unknown, cortisol half‐life in juvenile mink was typical for mammals (60 min; Sangild & Elnif, 1996). All individuals that were not anesthetized during sampling (*n* = 12) were removed from this analysis. ANCOVA was used to compare the slope of these relationships between lactating/nonlactating and resource‐limited/nonresource‐limited otters in a model that also included maternal age. Model residuals of GLMs were visually assessed for approximate normality, and residual plots were assessed for homoscedascity. For all tests, results were considered significant when *p *<* *0.05.

## 3 RESULTS

### 3.1 Glucocorticoid response to capture

The timing of the acute stress response to the capture event was assessed in all sea otters with known time between capture and anesthesia of ≤60 min (*n* = 85). Cortisol concentration increased with hold time (*F*_1,79_ = 8.80, *p* = 0.004). This effect was similar for both lactating and nonlactating females (ANCOVA, *F*_1,79_ = 0.01, *p* = 0.91) and was not influenced by resource limitation (*F*_1,79_ = 0.76, *p* = 0.39). Corticosterone levels did not increase with hold time (*F*_1,79_ = 0.01, *p* = 0.91). Across all samples, cortisol was the dominant GC released but was strongly correlated with corticosterone (Figure 5a). The ratio of cortisol:corticosterone averaged 11.8 ± 7.4 (SD). This ratio falls within the range reported as typical (10–100) for terrestrial mammals (Koren et al., 2012). Because independent effects were detected as potential drivers of the magnitude of GC release, we also evaluated the cortisol:corticosterone ratio as a response variable.

**Figure 3:**
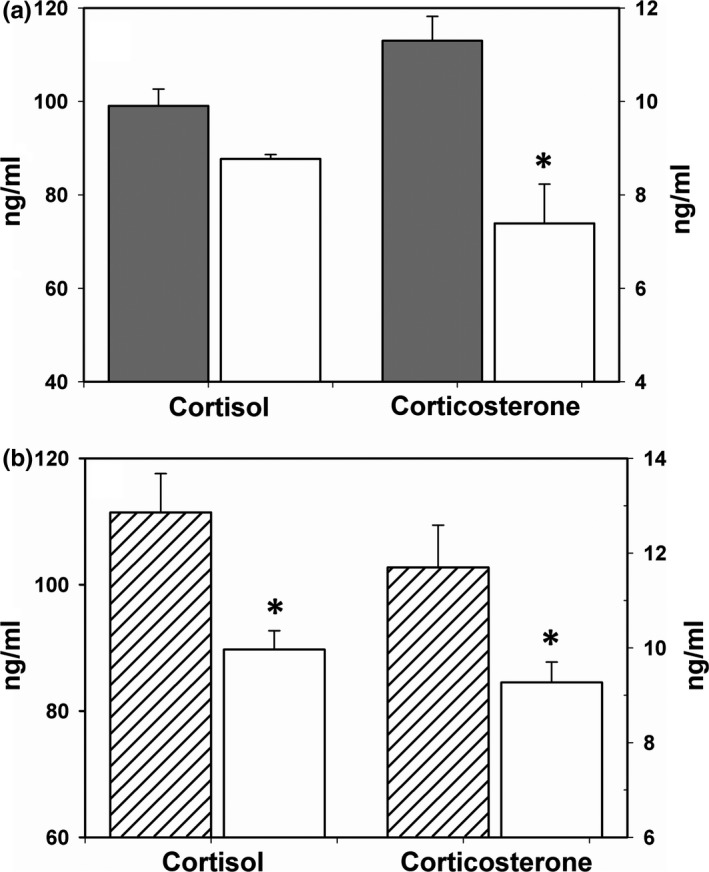
Alterations in the endocrine response from capture were evident during lactation and resource limitation. (a) Filled bars represent nonlactating females, open bars represent lactating females. (b) Filled bars represent pooled resource‐abundant females; open bars represent pool resource‐limited females. “*” denotes significance in least square means between lactation and nonlactating or resource‐abundant and resource‐limited sea otters from the general linear model

**Figure 4:**
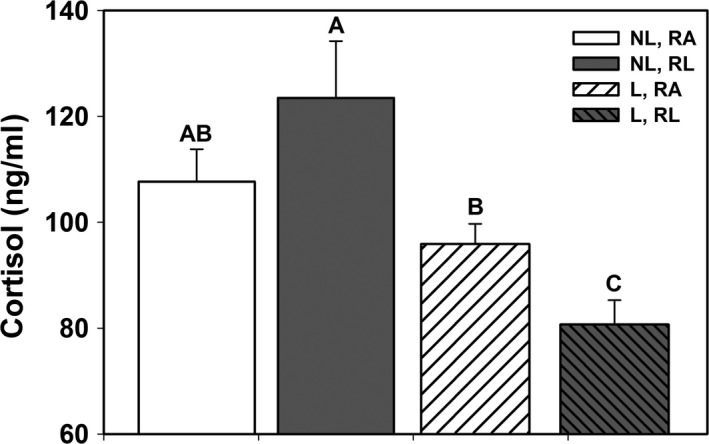
Suppression of cortisol release after capture was most apparent in lactating sea otters, and lowest under conditions of resource limitation. Bar shading denotes lactation status (lactating, L; nonlactating, NL) and resource limitation status (resource abundant, RA; resource limited, RL). Different letters denote differences in least square means from the GLM. Suppression of cortisol release during lactation was evident in resource‐limited otters

**Figure 5:**
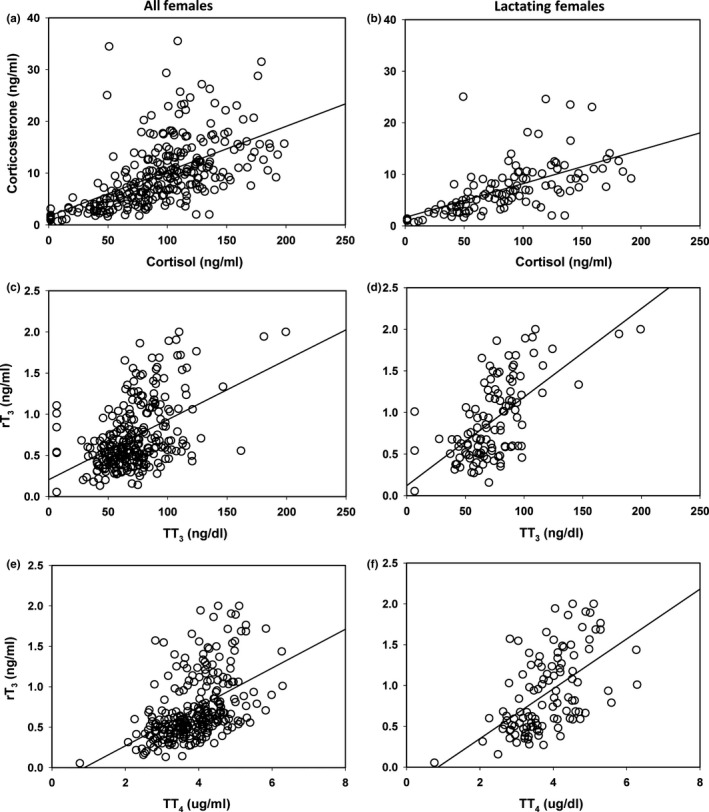
Interrelationships between measured analytes. (a) Relationship between cortisol and corticosterone of all female sea otters, *y* = 1.67 + 0.09*x*,* r* ^2^ = 0.33, *F*_1,276_ = 133.73, *p *<* *0.0001. (b) Relationship between cortisol and corticosterone for lactating females, *y* = 1.64 + 0.07*x*,* r* ^2^ = 0.35, *F*_1,110_ = 59.31, *p *<* *0.0001. (c) Relationship between T_3_ and rT_3_, *y* = 0.20 + 0.007*x*,* r* ^2^ = 0.22, *F*_1,272_ = 76.10, *p *<* *0.0001. (d) Relationship between T_3_ and rT_3_ in lactating females, *y* = 0.12 + 0.01*x*,* r* ^2^ = 0.38, *F*_1,110_ = 68.32, *p *<* *0.0001. (e) Relationship between T_4_ and rT3, *y* = −0.21 + 0.24*x*,* r* ^2^ = 0.24, *F*_1,264_ = 82.91, *p *<* *0.0001. (f) Relationship between T_4_ and rT_3_ in lactating females, *y* = −0.26 + 0.31*x*,* r* ^2^ = 0.31, *F*_1,108_ = 48.15, *p *<* *0.0001

GC concentrations after the capture stress response showed differences with lactation status and resource limitation, but not by subspecies. Nonlactating females had higher corticosterone concentrations than lactating females (Tables 1, 2; Figure 3a). Resource limitation was associated with a reduced magnitude of GC release for both GCs (Tables 1, 2; Figure 3b). There was a significant interaction of lactation status and resource limitation for cortisol release in response to capture (Table 2; Figure 4). Cortisol release was lower in resource‐limited sea otters and lactating females from resource‐abundant subpopulations exhibited a stronger acute stress response. Corticosterone release increased significantly with maternal age (Table 2). There were no differences between subspecies in the magnitude of GC release in response to capture (*p *>* *0.05). The cortisol:corticosterone ratio was strongly impacted by lactation status (*F*_1,256_ = 11.88, *p *<* *0.001), having a value of 10.3 ± 0.4 (SE) for lactating females and 14.0 ± 0.9 (SE) for nonlactating otters, but was not altered by any other explanatory factor (*p *>* *0.05). For all sea otters, corticosterone increased with cortisol concentrations (Figure 5a). The slope of this association was greater in nonlactating sea otters (ANCOVA, *F*_1,274_ = 4.2, *p* = 0.04; Figure 5b).

**Table 1:** Mean ± SE of analytes

	NSO	SSO	Nonlactating	Lactating	Resource‐abundant	Resource‐limited
TAC (mM)	0.56 ± 0.03*	1.09 ± 0.03*	0.80 ± 0.02	0.79 ± 0.04	0.87 ± 0.04	0.77 ± 0.02
IgM (mg/ml)	0.22 ± 0.02*	0.60 ± 0.02*	0.37 ± 0.02	0.43 ± 0.03	0.39 ± 0.03	0.30 ± 0.01
IgG (mg/ml)	17.10 ± 1.00*	23.79 ± 1.06*	18.35 ± 0.88	22.75 ± 1.38	17.73 ± 1.47ᵟ	20.77 ± 0.72ᵟ
Cortisol (ng/ml)	94.97 ± 4.21	94.58 ± 4.36	99.04 ± 3.61	87.68 ± 5.83	111.43 ± 6.16ᵟ	89.74 ± 2.98ᵟ
Corticosterone (ng/ml)	10.11 ± 0.61	9.51 ± 0.63	11.30 ± 0.52^#^	7.39 ± 0.84^#^	11.70 ± 0.89ᵟ	9.27 ± 0.43ᵟ
TT_4_ (ug/dl)	3.59 ± 0.08*	4.19 ± 0.08*	3.92 ± 0.06	3.75 ± 0.11	3.75 ± 0.11	3.89 ± 0.05
TT_3_ (ng/dl)	60.96 ± 2.28*	82.63 ± 2.40*	70.26 ± 2.00	71.65 ± 3.16	78.25 ± 3.36ᵟ	68.57 ± 1.62ᵟ
rT_3_ (ng/ml)	0.52 ± 0.03*	0.90 ± 0.03*	0.60 ± 0.02^#^	0.86 ± 0.04^#^	0.75 ± 0.04ᵟ	0.68 ± 0.02ᵟ

Note

TAC: total antioxidant capacity; TT_4_: total T_4_; TT_3_: total T_3_; rT_3_: reverse T_3_; NSO: northern sea otter; SSO: southern sea otter. Different superscripts denote significant differences between subspecies, lactation, or resource limitation status from a general linear model.

**Table 2:** Results from general linear model examining effects of subspecies, and lactation and resource‐limited status on serum or plasma analytes in sea otters

	TAC	IgM	IgG	Cortisol	Corticost	TT_4_	TT_3_	rT_3_
Den *df*	245	246	247	256	256	244	252	256
Subspecies								
F	221.90	289.62	31.52	0.16	1.76	44.01	58.63	138.44
P	**<0.0001** S > N	**<0.0001** S > N	**<0.0001** S > N	0.69	0.19	**<0.0001** S > N	**<0.0001** S > N	**<0.0001** S > N
Lactating								
F	0.43	2.07	3.42	0.002	12.52	1.52	2.08	56.83
P	0.51	0.15	0.07	0.96	**0.0005** NL > L	0.22	0.15	**<0.0001** L > NL
Resource‐limited								
F	1.72	0.43	4.57	15.91	5.23	2.09	8.68	9.13
P	0.19	0.51	**0.03** RL > RA	**<0.0001** RA > RL	**0.02** RA > RL	0.15	**0.004** RA > RL	**0.003** RA > RL
Maternal age								
F	5.10	1.18	2.08	0.75	11.31	1.94	0.66	8.58
P	**0.02** INCR	0.28	0.15	0.39	**0.0009** INCR	0.17	0.42	**0.004** DECR
RL × Lact								
F	0.52	1.81	1.45	5.12	0.62	0.07	0.80	2.44
P	0.47	0.18	0.23	**0.02**	0.43	0.80	0.37	0.12

Note

S: southern sea otter; N: northern sea otter; NL: nonlactating; L: lactating; RL: resource‐limited; RA: resource‐abundant; INCR: concentration increased with age; DECR: concentration decreased with age; TAC: total antioxidant capacity; TT_4_: total T_4_; TT_3_: total T_3_; rT_3_: reverse T_3_. Inequalities denote directions of significant differences.

### 3.2 Thyroid hormones

There was no relationship between time to sampling and the serum concentration of any thyroid hormones (T_4_, T_3_, rT_3_; *p *>* *0.05). T_4_ and T_3_, and rT_3_, all showed significant covariation with each other. T_3_ and rT_3_ showed a positive relationship (Figure 5c), and the slope of association was greater in lactating females (ANCOVA, *F*_1,270_ = 23.15, *p *<* *0.0001, Figure 5d). T_3_ and rT_3_ also increased with an increase in T_4_ (*r* ^2^ = 0.19, *F*_1,264_ = 62.49, *p *<* *0.0001; Figure 5e, respectively). The slope of association between rT_3_ and T_4_ was greater in lactating females (ANCOVA, *F*_1,262_ = 4.27, *p* = 0.04, Figure 5f).

There was a strong impact of subspecies on all three thyroid hormones (Tables 1, 2) with SSO having greater concentrations (T_4_: 17% higher, T_3_: 36% higher, rT_3_: 73% higher). There was no impact of lactation status on T_3_ or T_4_ (*p *>* *0.05) but mean rT_3_ concentration was 48% greater in lactating otters (Tables 1, 2). Resource limitation impacted both T_3_ and rT_3_ concentrations (Tables 1, 2). In resource‐limited sea otters, mean T_3_ and rT_3_ concentrations declined 12% and 9%, respectively. There were no significant interactions between lactation status and resource limitation for any thyroid hormone (*p *>* *0.05). Maternal age did not influence T_4_ or T_3_ concentrations (*p *>* *0.05). In contrast, rT_3_ concentrations decreased with maternal age (Table 2).

### 3.3 Immune markers

The two immune markers were weakly associated across samples. (*r* ^2^ = 0.19, *F*_1,266_ = 62.27, *p *<* *0.0001). Mean concentrations of immune markers, IgM and IgG, were 172% and 39% higher, respectively, in SSO than NSO (Table 1). There were no differences in immune markers between lactating and nonlactating otters (*p *>* *0.05; Tables 1, 2). Resource‐limited otters had higher IgG concentrations than resource‐abundant otters (Tables 1, 2). There was no evident interaction between lactation status and resource limitation for either immune marker (Table 2). Similarly, there was no influence of maternal age on either immune marker (Table 2).

### 3.4 Total antioxidant capacity

There was a strong difference in the TAC of the subspecies, with SSO exhibiting a mean TAC that was 89% greater than NSO (Tables 1, 2). There was no evident impact of lactation status, resource limitation, or their interaction on TAC (Tables 1, 2). There was a significant increase in TAC with maternal age (Tables 1, 2).

### 3.5 Associations between markers

**Figure 6:**
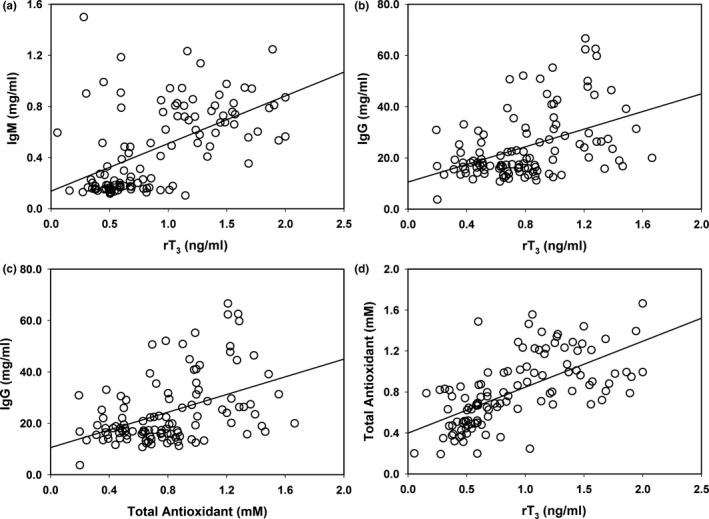
Interrelationships between measured analytes for lactating females. (a) Relationship between rT_3_ and IgM, *y* = 0.14 + 0.37*x*,* r* ^2^ = 0.29, *F*_1,109_ = 44.63, *p *<* *0.0001. (b) Relationship between rT_3_ and IgG, *y* = 13.66 + 11.77*x*,* r* ^2^ = 0.18, *F*_1,110_ = 24.64, *p *<* *0.0001. (c) Relationship between total antioxidant capacity and IgG, *y* = 10.57 + 17.21*x*,* r* ^2^ = 0.20, *F*_1,109_ = 26.44, *p *<* *0.0001. (d) Relationship between rT_3_ and total antioxidant capacity, *y* = 0.40 + 0.45*x*,* r* ^2^ = 0.41, *F*_1,109_ = 74.48, *P *<* *0.0001

We examined relationships among analytes of different categories (e.g., GCs vs. thyroid hormones) to determine possible covariation or suggested downstream effects on another marker. We included lactation status and an interaction term with lactation status in the model to assess whether the slope of the regression was similar between groups. T_4_ and T_3_ increased with cortisol (*F*_1,262_ = 14.83, *p *<* *0.0001; *F*_1,270_ = 13.69, *p* = 0.0003, respectively), and the slope of this association was similar among lactating and nonlactating otters. Both immune markers covaried with thyroid hormones. IgM and IgG increased with T_4_ (*F*_1,262_ = 28.38, *p *<* *0.0001; *F*_1,262_ = 8.84, *p* = 0.003, respectively), T_3_ (*F*_1,264_ = 46.78, *p *<* *0.0001; *F*_1,265_ = 7.41; *p* = 0.007, respectively) and rT_3_ (Figure 6a, *F*_1,264_ = 63.75, *p *<* *0.0001; Figure 6b, *F*_1,265_ = 20.80, *p *<* *0.0001, respectively). These associations were not affected by lactation status except for the effect of T_3_ on IgG which was greater in lactating otters (ANCOVA, *F*_1,265_ = 18.50, *p *<* *0.0001).

Increased TAC was associated with increased immunoglobulins and thyroid hormones. Both immunoglobulins showed a positive association with TAC (IgM: *F*_1,262_ = 101.13, *p *<* *0.0001; IgG: *F*_1,263_ = 31.14, *p *<* *0.0001). The slope of the increase in IgG with TAC was greater in lactating otters (ANCOVA, *F*_1,263_ = 14.39, *p* = 0.0002; Figure 6c). TAC increased with T_4_ (*F*_1,260_ = 38.58, *p *<* *0.0001), and the slope of this relationship was greater in lactating otters (ANCOVA, *F*_1,260_ = 6.30, *p* = 0.01). TAC also increased with T_3_ (*F*_1,263_ = 72.34, *p *<* *0.0001) and rT_3_ (Figure 6d) but no effects of lactation on the slopes of these associations were evident (ANCOVA, *p *>* *0.05).

## 4 DISCUSSION

### 4.1 Glucocorticoids

We measured the release of GCs in response to an acute capture stress. The wide individual variability in this response likely reflected both individual differences (e.g., personalities) but also the several factors that showed impacts on the magnitude of cortisol release. Cortisol was the predominant GC released, but sea otters also released biologically significant and varying quantities of corticosterone in response to acute stress.

A previous investigation in sea otters found that corticosterone was most responsive to capture stress (Larson, Monson, Ballachey, Jameson, & Wasser, 2009), and that levels of this GC may be affected by genetic diversity (Larson, Ralls, & Ernest, 2015). In the current study, corticosterone levels after capture were significantly decreased in lactating sea otters suggesting reduced HPA responsiveness to the acute capture stress. Although it might be expected that lactation status would be reflected by higher levels of baseline GCs, the prolonged energetic stress of lactation may have blunted the ability to mount an additional stress response to handling for corticosterone (Harlow et al., 1992; Krause et al., 2014; Rich & Romero, 2005), resulting in acclimation of the stress response (Juster, McEwen, & Lupien, 2010; McEwen, 2000; Wingfield & Mukai, 2009). The reduced corticosterone release in lactating females and the lack of a similar effect for cortisol resulted in increases in the cortisol: corticosterone ratios during lactation. Cortisol release was lower in resource‐limited otters, suggesting impacts of nutritional stress on HPA sensitivity. Resource limitation also interacted with the effect of lactation on cortisol release, with cortisol only showing lactation effects under conditions of resource limitation (Figure 4). Under lactation stress, females from subpopulations with abundant nutritional resources mounted a more pronounced acute stress response. Together, these results suggest a complex semi‐independent release of these two GCs with each one responding differently to varying types and levels of stressors while being modulated by individual genetic makeup and background environmental and physiological stress levels. Though limited, data from mammals suggest that GCs can vary independently showing seasonal variation, circadian patterns, and responses to acute stress (Vera, Antenucci, & Zenuto, 2011). Cortisol binding globulin (CBG) generally shows greater specificity for the dominant glucocorticoid (Westphal, 1986); thus, the two GCs may have different representation in the “free” bioavailable glucocorticoid pool and may have independent signaling and functional roles (Koren et al., 2012) including partitioning of physiological and behavioral effects.

### 4.2 Thyroid hormones

Thyroid hormones regulate metabolic pathways and metabolism. Decreases in maintenance metabolism may serve to increase the proportion of assimilated energy available for milk synthesis but reduce metabolic heat production to thermoregulation. Thyroid hormones were substantially higher in SSO than in NSO. This difference, assessed in a model that controlled for lactation status and nutritional limitation, suggests potential phenotypic differences among the two subspecies in energy requirements. This may reflect differences in habitat and resource availability during the divergence of the subspecies, or simply reflect differences created through bottlenecking and founder effects resulting from historic over exploitation of sea otters in the 18th and 19th centuries (Larson, Jameson, Enier, Fleming, & Bentzen, 2002; Larson, Jameson, Etnier, Jones, & Hall, 2012). Also, although our southern and northern samples had comparable numbers of nonresource‐limited individuals (SSO = 30, NSO = 31), it is worth noting that our sample of resource‐limited SSO otters (*n* = 107) came from subpopulations that had been at or near carrying capacity for much longer than most of the northern subpopulations we sampled. For example, our resource‐limited subpopulations in central California were among the first areas to recover from the fur trade in the early 1900s (Kenyon, 1969) and have been at or near carrying capacity for many decades (Tinker et al., 2017). Conversely, sea otter populations in both Washington and SE Alaska were established by re‐introduction in 1969 and 1970 (Jameson et al., 1982; Kenyon, 1969), and thus, our resource‐limited samples from these regions come from subpopulations that have only recently reached carrying capacity. Similarly, sea otters in western Prince William Sound are believed to have been at carrying capacity for several decades, although this population was depleted by the *Exxon Valdez* Oil Spill in 1989, and only recently has recovered to the point that resources are once again the primary limiting factor (Ballachey et al., 2014; Monson, Doak, Ballachey, & Bodkin, 2011; Monson, Doak, Ballachey, Johnson, & Bodkin, 2000).

Lactation had no effect on T_4_ or T_3_, suggesting that downregulation of maintenance metabolism is not accomplished by suppressing thyroid availability. However, rT_3,_ which should compete with T_3_ for receptor sites, increased by 86% in lactating females, which should downregulate metabolism. Similarly, T_3_ decreased in resource‐limited otters, suggesting downregulation of metabolism under stronger nutritional constraints. Sea otters under resource limitation stress may face intense prey competition, increased foraging effort (number of dives, dive length, and foraging bout duration), and still experience a negative energy balance (Thometz, Staedler, et al., 2016; Tinker et al., 2017). It may be adaptive for individuals under nutrient restriction to decrease thyroid levels to decrease metabolic rate, and thus energy requirements. More difficult to explain is the small decrease in rT_3_ in resource‐limited sea otters, although the difference is small (9%) and may reflect lower overall rates of deiodination under nutritional stress.

T_4_, T_3_, and rT_3_ all showed strong, positive associations with each other. T_4_ may act as a reservoir for downstream processes that may require conversion to T_3_ (for promotion of thermogenesis or muscle catabolism) or conversion to rT_3_ (for suppression of metabolic rate). T_4_ and T_3_ both weakly increased with cortisol after handling. If higher magnitude cortisol responses to handling are associated with lower baseline levels of stress, this pattern may reflect the reduced suppression of thyroid by baseline GCs (Ferguson & Peterson, 1992).

T_4_, T_3_, and rT_3_ showed positive associations with TAC, IgM, and IgG that were strongest in lactating females. This suite of associations suggests a role for thyroid in regulating energy mobilization for allocation toward immune and oxidative defense. Biomedical studies have shown strong links between variation in thyroid and tissue antioxidant responses (Das & Chainy, 2001; Venditti, Balestrieri, Di Meo, & De Leo, 1997). Similarly, thyroid variation has strong impacts on humoral immune responses (Fabris, 1973), mounting an immune response creates oxidative stress (Costantini & Møller, 2009), and animals may mitigate oxidative stress and enhance their response to immune challenges with increased dietary antioxidants (Catoni, Peters, & Schaefer, 2008; Park, Chyun, Kim, Line, & Chew, 2010). Together these associations may reflect a critical role for thyroid in regulating metabolism and allocation toward systemic defenses under the strong nutrient constraints of lactation.

### 4.3 Immune markers

Differences between subspecies were the strongest drivers of variation in circulating immunoglobulins. Immunoglobulin concentrations, especially IgM, were greater in SSO. Previous studies have shown dramatically higher exposure of SSO to some pathogens (e.g., *Leptospira* spp., *T. gondii*) (Hanni et al., 2003). Coastal urbanization and freshwater run‐off may result in increased pathogen exposure in SSO (Miller et al., 2002). Together, these findings suggest higher rates of pathogen exposure in SSO. Our findings suggest that immune responses to these pathogens are an important component of sea otter survival and are not downregulated in response to lactation. IgG concentrations were higher in resource‐limited subpopulations. This is consistent with earlier work that suggests that density‐dependent changes in prey choice and habitat use may increase sea otter pathogen exposure in the resource‐limited California coastal system (Johnson et al., 2009). The current study suggests self‐maintenance by mothers and that immune responses are not reduced under constraints of nutritional stress due to lactation or resource limitation.

### 4.4 Total antioxidant capacity

Contrary to our prediction, there were no differences in TAC due to lactation status. Females appear to balance any potential increases in ROS production induced by elevated energy expenditure or breath‐hold diving during lactation (Vázquez‐Medina et al., 2011) and the associated stressors correlated with pup care, which may be elevated under conditions of resource limitation. Females may compensate for increased ROS by endogenously producing more antioxidants or increasing antioxidant concentration by consuming a more diverse diet or switch to prey with higher antioxidant concentrations, as reflected in the maintenance of TAC despite the strong potential for increased oxidative stress. TAC was almost twofold higher in SSO. This difference may reflect a physiological response to increased ROS production due to increased breath‐hold diving for foraging differences between habitats. Alternatively, increased TAC may reflect increased consumption of antioxidants from prey. Mothers are required to spend significantly more time foraging to meet the energy demands of lactation and to supplement a growing pup (Esslinger et al., 2014; Thometz, Staedler, et al., 2016). In addition, dietary diversity tends to increase under conditions of resource limitation (Newsome et al., 2015). This may enhance the supply of exogenous antioxidants available through alternate prey types and reduce the need for antioxidant enzyme synthesis. Measurement of antioxidant enzyme concentrations is necessary to differentiate between these two possibilities. Similarly, measures of oxidative damage are necessary to know whether this increased antioxidant capacity is sufficient to prevent oxidative stress. In either case, our data do not suggest loss of antioxidant capacity or enhanced oxidative stress during reproduction in sea otters. Maternal age had a positive effect on antioxidant capacity. This may reflect enhanced diving or foraging efficiency with larger body size or increased experience, which may reduce the potential for oxidative damage in older females.

Our data suggest that lactation has significant effects on physiological functions in female sea otters. Evidence of blunted stress responses and downregulated thyroid function in lactating sea otters suggest fitness costs to current reproduction, especially in populations experiencing resource limitation. These alterations may lessen the ability of lactating females to respond to additional stressors such as injury or infection and may increase risk of ELS, a significant cause of mortality in California, attributed to >50% of the deaths of stranded, reproductively mature females examined between 2005 and 2012 (Chinn et al., 2016). Resource limitation may require more pronounced metabolic alterations during lactation to balance reproductive demands with those for survival, further exacerbating ELS. In top‐predator species that functionally modulate species composition within their environment and where prey resources are often a significant limiting factor, physiological trade‐offs during lactation may have downstream effects on parental care and life‐history decisions ultimately manifest in population‐level consequences (i.e., a high proportion of prime‐age reproductively active females succumb to ELS), which likely mediate predator–prey dynamics and affect food‐web stability. These population‐level and ecosystem effects are even more likely for income‐breeding strategists, where reproductive effort is directly linked to energy acquisition during lactation.

## CONFLICT OF INTEREST

None declared.

## AUTHOR CONTRIBUTIONS

All authors conceived the ideas and designed methodology. SMC, MTT, DHM, and MMS collected the data. SMC conducted laboratory analyses. SMC and DEC analyzed the data. SMC led the writing of the manuscript. All authors contributed critically to the drafts and gave final approval for publication.

## DATA ACCESSIBILITY

Data available from the Dryad Digital Repository: https://doi.org/10.5061/dryad.mk59gk0.
