# Supplementary material for: Lactation and resource limitation affect stress responses, thyroid hormones, immune function, and antioxidant capacity of sea otters (Enhydra lutris)
Source: Ecol Evol. 2018 Jul 25;8(16):8433–47. doi: 10.1002/ece3.4280 (PMC6145021; doi:10.1002/ece3.4280)
Supplement: Supplementary file 1 [file ECE3-8-8433-s001.docx]

**Appendix**

Table S1. Summary of metrics used to assess population status with respect to food resource abundance for 11 sea otter subpopulations in the northeast Pacific: ranging north to south these are west Alaska peninsula (WAP), east Alaska peninsula (EAP), Prince William Sound (PWS), northern Southeast Alaska (NSE), Washington State (WA), Elkhorn Slough (ESL), Monterey peninsula (MON), Big Sur (BSR), San Luis Obispo (SLO), Santa Barbara Channel (SBC) and San Nicolas Island (SNI): refer to Figure 2 for a map of these locations. For each sub-population (columns) a summary of published findings for each metric (rows, explanations in Column 1) is provided, with numbered references below each entry indicating the citations for further details of these findings. At bottom of the table a summary of inferred status is provided based on all available data for all metrics: RA = resources abundant, RL = resources limiting to growth.

|  | **Subpopulation** | | | | | | | | | | |
| --- | --- | --- | --- | --- | --- | --- | --- | --- | --- | --- | --- |
| **Metric** | WAP | EAP | PWS | NSE | WA | ESL | MON | BSR | SLO | SBC | SNI |
| Population growth: generally rapid when food resources abundant, slow as resources become limiting, stable at carrying capacity | declining^1^ 12 | rapid 12, 19 | slow 12 | slow 16,20 | slow^2^ 13 | rapid 7,23 | stable 6,7 | stable 6,7 | declining 5,7 | rapid 1,3,7 | rapid 1,2,7 |
| Population density: generally food resources abundant at low density, resources become limiting at high density | low 11, 12 | high 11, 12, 19 | high 11, 12 | low 11, 16, 20 | high^2^ 14 | medium 7,23 | high 6,7 | high 6,7 | high 5,7 | low 1,3,7 | low 2,7 |
| Diet diversity: generally low when food resources abundant, higher as resources become limiting and low quality prey added to diet | low 11, 12 | high 11, 12, 19 | high 11, 12 | high 11 | high 11, 21,22 | low 22,24 | high 6,8,11 | high 6,11 | high 2,8,11 | low 1,11 | low 2,8,11 |
| Prey/energy recovery rates while feeding: high when resources abundant, lower as resources become limiting | high 4,12 | medium 4,12, 19 | low 12 | low 16 | NA | high 22,24 | low 1,6 | low 1,6 | low 1,2,6 | high 1 | high 1,2,8 |
| Time-Activity budgets: low proportion of time budgets spent feeding (<40%) when resources abundant, high proportion of time spent feeding (>40%) when resources limiting | low 12 | low 12 | high 12 | high 16 | NA | low 22 | high 1,6,9 | high 1,6,9 | high 2,9 | low 1,9 | low 2,9 |
| Relative body condition: generally good body condition (high residuals from mass-length or mass-age funcitons) when resources abundant, poor condition as resources become limiting | good 12, 17, 18 | good 12, 17 | poor 12, 17 | poor 12, 17 | poor^2^ 22 | good 1,22 | medium 1,6 | poor 1,6 | poor 1,2 | good 1 | good 1,2 |
| **Status of Population based on combined metrics:** | **RA** | **RA** | **RL** | **RL** | **RL** | **RA** | **RL** | **RL** | **RL** | **RA** | **RA** |

^1^Population decline due to top-down forces (killer whale predation) and not bottom-up forces (prey became abundant as population declined).

^2^Samples for Washington were collected in the high-density, central portion of the range, not the low density range periphery where growth rate is higher.

**Literature Cited for Appendix**

1. Tinker, M.T., Tomoleoni, J., LaRoche, N., Bowen, L., Miles, A.K., Murray, M., Staedler, M. & Randell, Z. (2017) Southern sea otter range expansion and habitat use in the Santa Barbara Channel, California. US Geological Survey.
2. Tinker, M.T., Bentall, G. & Estes, J.A. (2008) Food limitation leads to behavioral diversification and dietary specialization in sea otters. *Proceedings of the National Academy of Sciences,* 105, 560-565.
3. Lafferty, K.D. & Tinker, M.T. (2014) Sea otters are recolonizing southern California in fits and starts. *Ecosphere,* 5, 1-11.
4. Tinker, M.T. (2015) The use of quantitative models in sea otter conservation. *Sea Otter Conservation*, pp. 257-300. Elsevier.
5. Tinker, M.T., Doak, D.F., Estes, J.A., Hatfield, B.B., Staedler, M.M. & Bodkin, J.L. (2006) Incorporating diverse data and realistic complexity into demographic estimation procedures for sea otters. *Ecological Applications,* 16, 2293-2312.
6. Tinker, M., Jessup, D., Staedler, M., Murray, M., Miller, M., Burgess, T., Bowen, E., Miles, K., Tomoleoni, J. & Thometz, N. (2013) Sea otter population biology at Big Sur and Monterey California: investigating the consequences of resource abundance and anthropogenic stressors for sea otter recovery. *Review. California Coastal Conservancy and the US Fish and Wildlife Service, Final Report. Santa Cruz, CA*.
7. Tinker, M.T. & Hatfield, B.B. (2017) California sea otter (Enhydra lutris nereis) census results, Spring 2017. US Geological Survey.
8. Tim Tinker, M., Guimarães, P.R., Novak, M., Marquitti, F.M.D., Bodkin, J.L., Staedler, M., Bentall, G. & Estes, J.A. (2012) Structure and mechanism of diet specialisation: testing models of individual variation in resource use with sea otters. *Ecology Letters,* 15, 475-483.
9. Thometz, N., Staedler, M., Tomoleoni, J., Bodkin, J.L., Bentall, G. & Tinker, M.T. (2016) Trade-offs between energy maximization and parental care in a central place forager, the sea otter. *Behavioral Ecology*, 27, 1552-1566.
10. Newsome, S.D., Tinker, M.T., Monson, D.H., Oftedal, O.T., Ralls, K., Staedler, M.M., Fogel, M.L. & Estes, J.A. (2009) Using stable isotopes to investigate individual diet specialization in California sea otters (*Enhydra lutris nereis*). *Ecology,* 90, 961-974.
11. Newsome, S.D., Tinker, M.T., Gill, V.A., Hoyt, Z.N., Doroff, A., Nichol, L. & Bodkin, J.L. (2015) The interaction of intraspecific competition and habitat on individual diet specialization: a near range-wide examination of sea otters. *Oecologia,* 178, 45-59.
12. Estes, J.A., Bodkin, J. & Tinker, M. (2010) Threatened southwest Alaska sea otter stock: delineating the causes and constraints to recovery of a keystone predator in the North Pacific Ocean. *North Pacific Research Board Final Report,* 717, 117.
13. Jeffries, Steven, Deanna Lynch, and Sue Thomas. 2016. Results of the 2015 Survey of the Reintroduced Sea Otter Population in Washington State. Interim Progress Report, Cooperative Agreement Number F13AC00287. Washington Department of Fish and Wildlife
14. Laidre, K.L., Jameson, R.J., Jeffries, S.J., Hobbs, R.C., Bowlby, C.E. & VanBlaricom, G.R. (2002) Estimates of carrying capacity for sea otters in Washington state. *Wildlife Society Bulletin*, 1172-1181.
15. Chinn, S.M., Miller, M.A., Tinker, M.T., Staedler, M.M., Batac, F.I., Dodd, E.M. & Henkel, L.A. (2016) The high cost of motherhood: end-lactation syndrome in southern sea otters (*Enhydra lutris nereis*) on the Central California Coast, USA. *Journal of Wildlife Diseases,* 52, 307-318.
16. Bodkin, J.L., Monson, D.H. & Esslinger, G.G. (2007) Activity budgets derived from time–depth recorders in a diving mammal. *Journal of wildlife management,* 71, 2034-2044.
17. Monson, D.H. (2009) *Sea otters (Enhydra lutris) and Steller sea lions (Eumetopias jubatus) in the North Pacific: Evaluating mortality patterns and assessing population status at multiple time scales*. University of California, Santa Cruz.
18. Laidre, K., Estes, J., Tinker, M., Bodkin, J., Monson, D. & Schneider, K. (2006) Patterns of growth and body condition in sea otters from the Aleutian archipelago before and after the recent population decline. *Journal of Animal Ecology,* 75, 978-989.
19. Coletti, H.A., Bodkin, J.L., Monson, D.H., Ballachey, B.E. & Dean, T.A. (2016) Detecting and inferring cause of change in an Alaska nearshore marine ecosystem. *Ecosphere,* 7.
20. Esslinger, G. & Bodkin, J. (2009) Trends in Southeast Alaska sea otter populations; 1969–2003. *USGS Scientific Investigations Report,* 5045, 18.
21. Laidre, K.L. & Jameson, R.J. (2006) Foraging patterns and prey selection in an increasing and expanding sea otter population. *Journal of mammalogy,* 87, 799-807.
22. USGS, unpublished data
23. Estes, J.A. & Tinker, M.T. (2017) Rehabilitating sea otters: Feeling good versus being effective; Chapter 20.
24. Hughes, B.B., Eby, R., Van Dyke, E., Tinker, M.T., Marks, C.I., Johnson, K.S. & Wasson, K. (2013) Recovery of a top predator mediates negative eutrophic effects on seagrass. *Proceedings of the National Academy of Sciences,* 110, 15313-1531.
